# Microstructure and Mechanical Properties of Ti + N Ion Implanted Cronidur30 Steel

**DOI:** 10.3390/ma12030427

**Published:** 2019-01-30

**Authors:** Jie Jin, Wei Wang, Xinchun Chen

**Affiliations:** 1 State Key Laboratory of Tribology, Tsinghua University, Beijing 100084, China; ion_beam_tech@163.com; 2 School of Mechanical, Electronic and Control Engineering, Beijing Jiaotong University, Beijing 100044, China; 3 School of Metallurgy Engineering, Xi’an University of Architecture and Technology, Xi’an 710055, China

**Keywords:** ion implantation, Cronidur30 steel, nanohardness, nanostructure, friction

## Abstract

In this study, Ti + N ion implantation was used as a surface modification method for surface hardening and friction-reducing properties of Cronidur30 bearing steel. The structural modification and newly-formed ceramic phases induced by the ion implantation processes were investigated by transmission electron microscopy (TEM), X-ray photoelectron spectroscopy (XPS), and grazing incidence X-ray diffraction (GIXRD). The mechanical properties of the samples were tested by nanoindentation and friction experiments. The surface nanohardness was also improved significantly, changing from ~10.5 GPa (pristine substrate) to ~14.2 GPa (Ti + N implanted sample). The friction coefficient of Ti + N ion implanted samples was greatly reduced before failure, which is less than one third of pristine samples. Furthermore, the TEM analyses confirmed a trilamellar structure at the near-surface region, in which amorphous/ceramic nanocrystalline phases were embedded into the implanted layers. The combined structural modification and hardening ceramic phases played a crucial role in improving surface properties, and the variations in these two factors determined the differences in the mechanical properties of the samples.

## 1. Introduction

Owing to its good corrosion resistance and mechanical performance as compared with 52100, M50, 9Cr18 or M50NiL bearing steels [1], Cronidur30 is increasingly applied for use in aerospace bearings [2,3]. Cronidur30 can be well reinforced based on nitrogen-based solid-solution strengthening, precipitation strengthening, and other similar processes to improve the hardness, corrosion resistance, wear resistance, and yield strength [4]. However, high nitrogen content can also induce inner defects like segregation and precipitation, leading to an increase in particle size and the formation of nonuniform granular carbon nitrides [5]. Moreover, the surface of Cronidur30 steel will usually suffer nitrogen loss during heat treatment or mechanical processing, which leads to low fracture elongation, brittleness behavior, and high sensitivity to surface stress corrosion cracking [6]. Therefore, surface modification techniques are required to improve the mechanical properties of high-nitrogen bearing steel, as well as its friction and wear performances. In recent years, ceramic coatings have been widely studied and used in harsh operational conditions on account of their high hardness, low wear, and chemical inertness [7]. However, the major drawback limiting the wide applicability of these coatings is probably due to their high contact stress and adhesive problems [8,9].

It is well known that ion implantation is attractive because of its unique merits [10]. One unique merit is that this method is free from adhesive problems and is capable of changing the grain size of the original components when the ions are implanted into the matrix [11]. A further merit is that the high-energy ions not only modify structures and components of the implanted subsurface at tens of nanometers, but also impact the zone located underneath the implanted subsurface by long-range effect [12,13]. Furthermore, it has been reported in many cases that the irradiation of a material by two or more distinct types of ions (for example, gas and metal ions) results in more significant changes in the properties of the material [14,15]. Implanted elements such as Cr, Ti, and N can undergo different degrees of phase formation and microstructural change, as well as mechanical and chemical enhancements [16,17,18,19,20,21,22]. For instance, Pogrebnjak found that an increase in hardness and a reduction of wear in double-implanted Ti alloys was attributed to the formation of small dispersion nitride, carbonitride, and intermetalloid phases [17]. Sudjatmoko highlighted that the optimum enhancement of hardness properties and wear resistance of Ti-6Al-4V alloy after nitrogen implantation was principally due to the formation of ceramic Ti_2_N and TiN phases [20]. Therefore, by investigating the different tribological behaviors of the two phases, including carbides and nitrides formed in the subsurface, especially in an oil-free lubrication environment, it is possible to prepare a low-friction and wear-resistant coating for high temperature or vacuum applications.

Due to the saturation state of nitrogen in the metallurgical equilibrium, it is difficult to further increase the concentration of nitrogen in Cronidur30 steel, especially after reaching 0.36% under the metallurgical equilibrium, and the metallurgical formation of nitride is insufficient when the steel surface is implanted with only nitrogen. The formation of more MN (M stands for metal) compounds can be more effective in improving mechanical properties than single metal element implantation [14]. Co-implantation of N and Ti ions has been reported to effectively improve mechanical and tribological properties of high-chromium cast iron alloy [16]. It has been noted that the presence of Ti after implantation can form defects such as dislocation defects and solid solution interstitials, resulting in improved mechanical properties [23]. Meanwhile, the implantation of nitrogen can effectively suppress the corrosion phenomenon in high-carbon steel, which is generally caused by carbon-related phases. Until now, little research has reported on the improvement of mechanical properties of Cronidur30 with Ti + N ion implantation, and there is currently no study that has looked at the strengthening mechanism. Thus, to meet the demand for improving mechanical performances of Cronidur30 bearing steel as mentioned above, Ti and N ion implantation will be proposed in this study. As indicated by the results, surface nanohardness enhancement and friction reduction of Cronidur30 bearing steel can be well tailored by controlling the growth of subsurface nanostructures including amorphous and ceramic nanocrystalline phases.

## 2. Experimental

### 2.1. Ion Implantation and Characterization

The circular discs made of Cronidur30 steel (containing 0.33 wt.% C, 0.8 wt.% Si, 0.41 wt.% Mn, 15.6 wt.% Cr, 0.25 wt.% Ni, and 0.93 wt.% Mo) [1] were used as substrates for the ion implantation process. These discs, with a diameter of 5 mm and a thickness of 10 mm, were grounded and polished with tungsten carbide (WC) abrasive paste (obtaining a surface roughness R_a_ = 20 nm). Prior to the ion implantation process, they were cleaned ultrasonically in acetone and ethanol for 10 min, respectively. These pristine samples were noted as S0 in this study.

**Figure 1:**
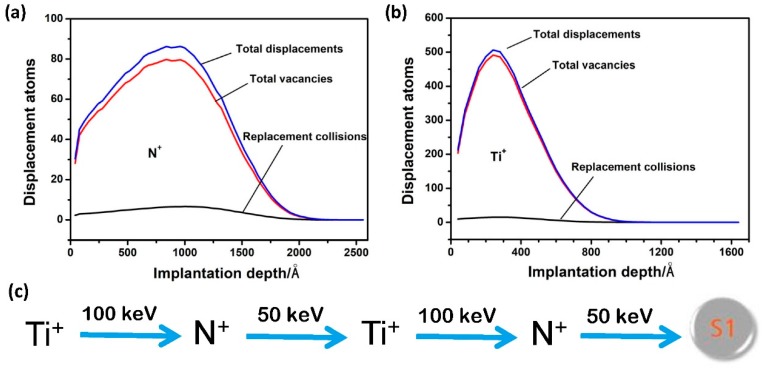
Ion implantation processes: (**a**,**b**) Transport of Ions in Matter (TRIM) simulation results showing the N^+^ and Ti^+^ implantation-induced distribution of displacement atoms at fluence of 1.0 × 10^17^ ions/cm^2^ as a function of implantation depth, respectively, and (**c**) processing parameters of dual Ti + N implanted sample S1.

Two kinds of ions, Ti^+^ and N^+^, were produced by metal evaporation vacuum arc (MEVVA) ion source (Ti target with 99.9% purity) and electron cyclotron resonance (ECR) gas ion source (N_2_ gas with 99.99% purity), respectively, for ion implantation. The parameters for implantation are listed as follows: Base pressure of 3.0 × 10^−4^ Pa and sample temperature of 25 to 40 °C. The average ion energies for Ti^+^ and N^+^ were 100 keV and 50 keV, respectively, which were chosen for the purpose that the ion implantation energy was sufficient for inducing significant structural strengthening but avoiding excessive irradiation damage to the steel surface. As shown schematically in Figure 1, the dual Ti + N implanted samples (noted as S1) were prepared by alternative implantation for 2 cycles. Before implantation, a rough calculation of the choice of ion influence was performed. According to the program of Transport of Ions in Matter (TRIM) simulation results, the maximum implantation depths of N^+^ and Ti^+^ ions at the fluence of 1.0 × 10^17^ ions/cm^2^ were about 200 nm and 100 nm, respectively. Assuming a Gaussian distribution, the average implantation depths of the N^+^ and Ti^+^ ions were roughly expected to be 100 nm and 50 nm, respectively, which provided reasonable space for the structural alteration and phase transformation. This speculation is almost consistent with the experimental result as observed in the S1 sample (depth of ~80 nm for the ion-implanted layer, which will be discussed below). Therefore, the total fluence for dual elements was set at 2 × 10^17^ ions/cm^2^, in which the Ti^+^ or N^+^ was implanted with a total fluence of 1.0 × 10^17^ ions/cm^2^ and 0.5 × 10^17^ ions/cm^2^ at each step. The final surface roughness R_a_ of S1 sample after ion implantations is 24 nm, as smooth as the pristine surface.

Surface roughness was examined with a three-dimensional white-light interference surface topography instrument (NexView 3D Optical Surface Profiler, ZYGO, Connecticut, USA). The cross-sectional structures of the samples were examined using a high-resolution transmission electron microscopy (HRTEM, JEM 2010F, JEOL, Tokyo, Japan) with a resolution of 0.108 nm and an attached energy-dispersive X-ray spectroscopy (EDS, Oxford Instruments, Oxfordshire, UK). The components of the implanted zone were examined by X-ray photoelectron spectroscopy (XPS). A monochromatic Al X-ray source was used with Al Kα line energy at about 1486.6 eV (PHI Quantera SXM, ULVAC-PHI, Kanagawa, Japan). The incident angle of the X-rays was 45 degrees. To examine the elemental distribution vs. depth, a sputtering argon gun was used for etching. The accelerating voltage was 4 kV, and the etching rate for a standard SiO_2_/Si sample was 5 nm/min. The crystalline structure of the surface layer was examined by a glancing incidence X-ray diffraction (GIXRD) method. CuKα radiation of 0.1541882 nm was used (D8 Advance, Bruker, Massachusetts, USA). The GIXRD measurements were performed at beam incidence angle of 1 degree. The nanohardness of each sample was tested with a Berkovich indenter using 10-mN maximum load. The hardness values were determined with mean value from 6 measurements.

### 2.2. Tribological Properties

The tribological tests were performed at room temperature (20 to 25 °C) in a ball-on-disc configuration (CETR-UMT-3, Bruker, Massachusetts, USA) with Si_3_N_4_ balls (d = 4 mm) as counterparts. The applied load was 2 N, and the frequency was 2 Hz. Two samples were used for testing: S0 and S1. Each test was repeated at least 3 times in order to obtain reliable results. 

## 3. Results

### 3.1. Surface Structure and Composition Evaluation

#### 3.1.1. HRTEM Analysis

**Figure 2:**
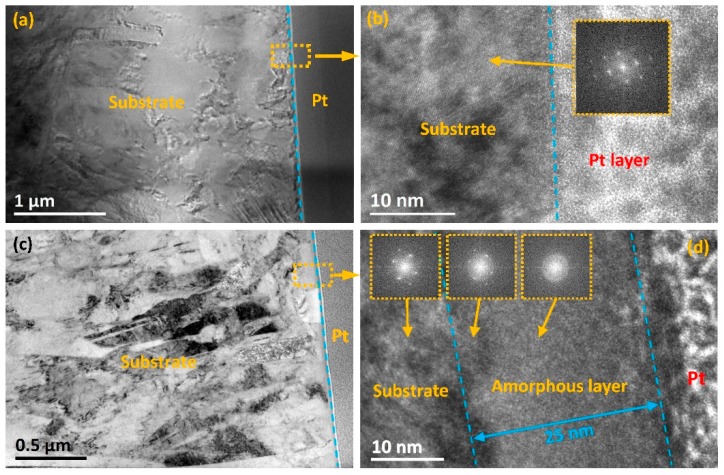
Transmission electron microscopy (TEM) cross-sectional images of (**a**,**b**) S0, (**c**,**d**) S1.

Figure 2 shows the cross-sectional TEM images of Cronidur30 (S0) and Ti + N dual implanted sample (S1). The protective Pt layer, which is used to prepare thin TEM samples, is composed of amorphous phases. The HRTEM image of S0 reveals that the lattice fringes of body-centered cubic (bcc) martensite appear in an ordered manner, indicating that it had a near-perfect crystalline structure (Figure 2b). However, after ion implantation, an additional amorphous sublayer could be seen at a depth of approximately 20 to 25 nm (Figure 2c,d) beneath the steel surface in the cases of S1. The length of this amorphous sublayer was larger than the case of co-implantation of Ti + Cr elements [23]. Moreover, as shown in Figure 2c, after Ti + N ions were implanted into S0, the high-density dislocations and twins nucleated and agglomerated near the amorphous sublayer, which led to gradually refined lath martensite grains along the implanted layer [24]. The HRTEM image of the refined sublayer in Figure 2d reveals that the lattice fringes were slightly disordered, indicative of crystal defects. Specifically, the closer to the amorphous sublayer, the less the diffraction rings as well as the darker the diffraction spots. As a whole, the amorphous sublayer mainly consisted of a nanocrystalline phase and an amorphous phase, wherein the crystal planes were disordered and the degree of atom arrangement was low. The presence of nanocrystalline structure or the amorphous phase is consistent with the broadened halo observed in the selected area electron diffraction pattern in Figure 2d. This amorphous sublayer is expected to reduce friction and wear more extensively [25].

**Figure 3:**
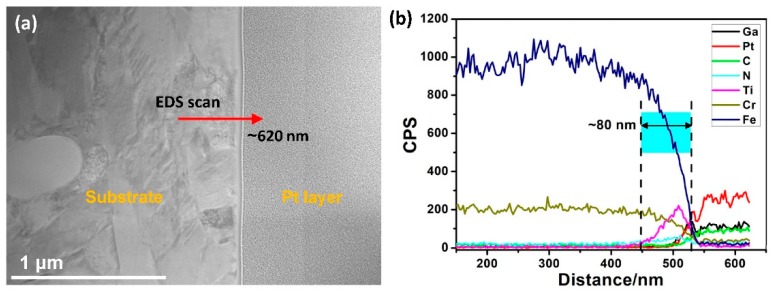
Results of TEM–EDS line analysis of S1: (**a**) TEM image showing the energy-dispersive X-ray spectroscopy (EDS) scan position and (**b**) elemental intensity profiling as a function of scan distance.

Figure 3 shows the EDS line scan results for sample S1. It is obvious that the implanted Ti^+^ ions diffused as deep as 80 nm for S1, even though the amorphous sublayer only had a shallow depth of 20 to 25 nm (Figure 2). Clearly, a secondary sublayer formed beneath the amorphous sublayer. As per the TEM results, this layer was mostly crystallized, referred to here as implanted-crystal sublayer. In order to elucidate the relationship between the elemental distribution and the bonding states of the implanted ions, XPS analysis was performed, as described below.

#### 3.1.2. XPS Analysis

**Figure 4:**
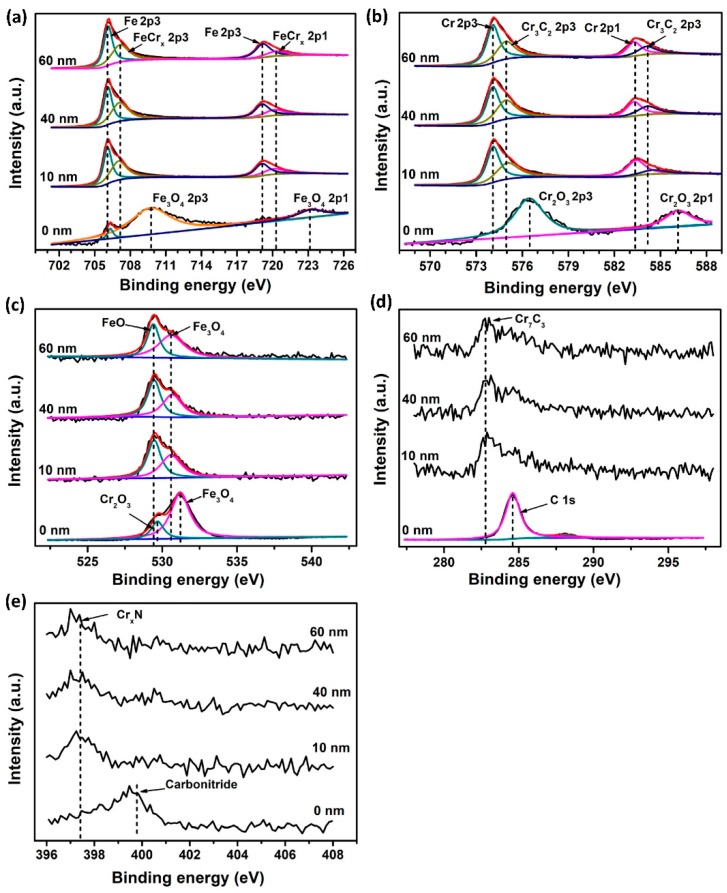
(**a**) Fe 2p, (**b**) Cr 2p, (**c**) O 1s, (**d**) C 1s, and (**e**) N 1s core energy level spectra of S0 at different sputtering depths (0, 10, 40, and 60 nm).

XPS depth profiling results of S0 are displayed in Table 1 and Figure 4. It is noted that the main chemical components of outer-most surface (depth = 0 nm) were different from that of inner layers (10, 40, 60 nm). The top surface of S0 mainly consisted of Fe_3_O_4_, Cr_2_O_3_ and carbonitride due to the surface oxidation and contaminants. In comparison, the inner layer was composed of Fe, FeCr_x_ alloys, CrC_x_ and Cr_x_N. Meanwhile, a small amount of Fe_3_O_4_ was also present. From Table 1, it can be seen that oxygen concentration dramatically decreased from the top surface to the inner layer. Correspondingly, the content of oxides was greatly reduced or even disappeared. Moreover, for the inner layer, the chemical states and concentrations of these elements (C, N, O, Cr, and Fe) were nearly constant as a function of depth. According to the overwhelming peak intensity (Figure 4) and dominant atomic concentration (Fe and Cr, Table 1), it was concluded that the chemical bonds formed in the bulk of S0 were mainly Fe–Fe and Cr–Cr bonds.

**Figure 5:**
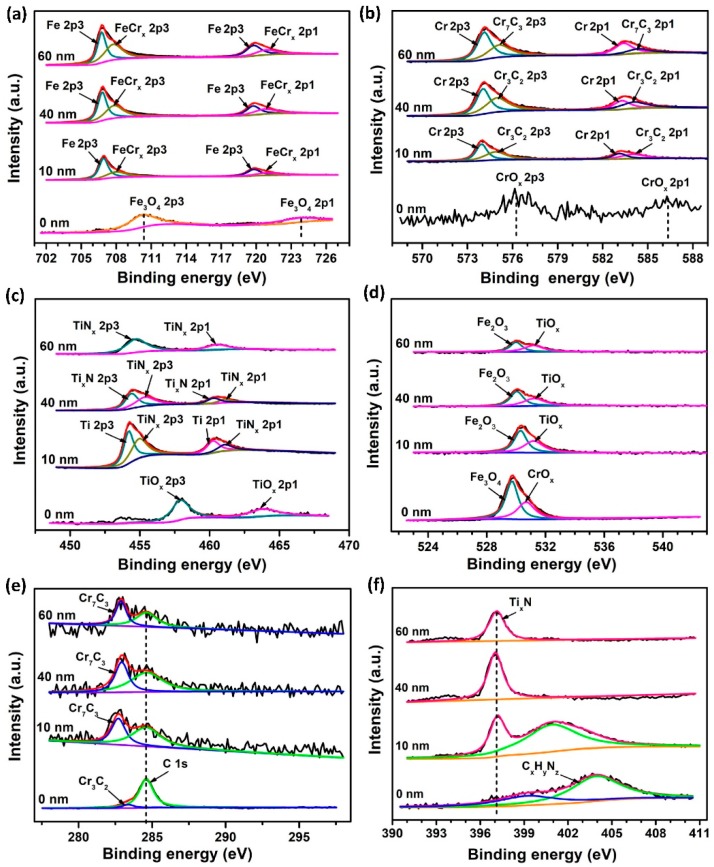
(**a**) Fe 2p, (**b**) Cr 2p, (**c**) Ti 2p, (**d**) O 1s, (**e**) C 1s, and (**f**) N 1s core energy level spectra of S1 at different sputtering depths (0, 10, 40, and 60 nm).

XPS results of the sublayers in the Ti + N implanted sample S1 are displayed in Table 2 and Figure 5. It is noted that the main chemical components of the outer-most surface (0 nm) were also different from that of inner layers (10, 40, 60 nm). The chemical components in the top surface of S1 were mainly Fe_3_O_4_, CrO_x_, TiO_x_, and carbonitride. In addition, the chemical states of Fe 2p, Cr 2p, and Ti 2p at the depth of 10 nm strongly indicate that the major chemical components of the amorphous layer in S1 were chromium carbides and titanium nitrides. Furthermore, according to the evolution of chemical states of Ti2p in S1, the main bonding types of Ti in the implanted-crystal layer were Ti - N compounds after N was implanted in S1. Another noticeable finding is that the carbon content on S1 surface (depth = 0 nm) was still very high (51.13 at.%) even after the pre-cleaning process before the ion implantation. Thus, the origin of this high carbon content was different from the case in So sample (51.55 at.%). We speculate that this abnormal enrichment level of carbon could be attributed to the beam-induced carbon uptake into the targeted surface even under an ultrahigh vacuum. The details regarding this influence are discussed below.

#### 3.1.3. GIXRD Analysis

**Figure 6:**
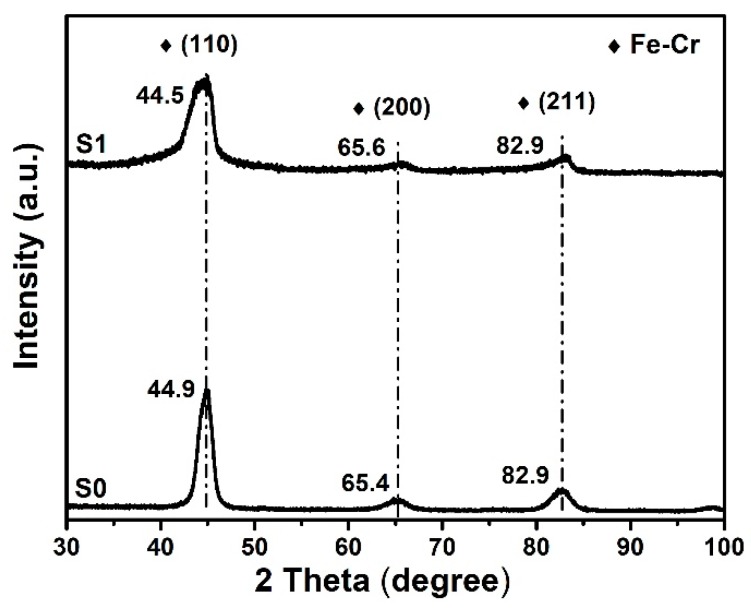
Grazing incidence X-ray diffraction (GIXRD) spectra of samples S0, S1.

A comparison of GIXRD data of samples S0 and S1 (Figure 6) shows that their phase structure mainly consisted of solid solution of cubic Fe–Cr, which grew preferentially along Fe–Cr (110) orientation. As compared to the main peak of Fe–Cr (110) in S0, the one measured in S1 slightly shifted to a lower angle. This phenomenon could originate from the combination of the residual compressive stresses induced by ion implantation and the expansion of crystal lattices caused by incorporating foreign ions [26].

As shown in Table 3, the grain size decreased after ion implantation, which is consistent with the broadened diffraction peaks observed in Figure 6. Furthermore, an increase in the FWHM of these peaks was observed and may result from the small size of the coherency domains and second-order stresses (distortion, shear of the lattices) inside the grains [27]. This phenomenon confirms the amorphization trend of the chromium carbides and titanium nitrides observed from XRD patterns, but hardly detected in the XPS spectra.

### 3.2. Mechanical Properties

#### 3.2.1. Nanohardness

**Figure 7:**
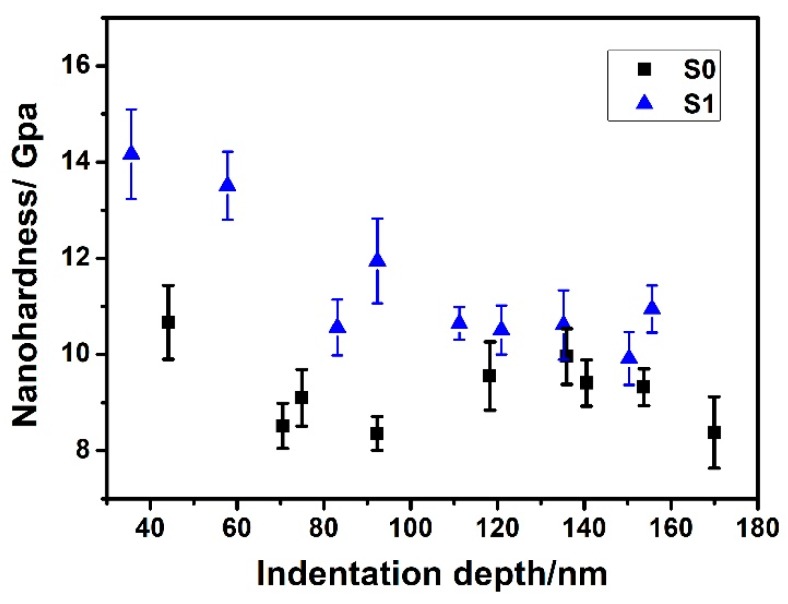
Nanohardness versus indentation depth for samples S0, S1.

The nanohardness curves of S0 and S1 are shown in Figure 7. The nanohardness decreased from ~10.5 to ~8.5 GPa when the indentation depth varied from ~40 to ~170 nm for S0. For the ion-implanted sample S1, it showed a similar trend as that of S0. However, the average hardness of S1 measured at each indentation depth was higher than that of S0. Furthermore, the nanohardness curves exhibited a rise at 90 nm and 120 nm, respectively. As seen in Figure 7, the maximum values of hardness of S0 and S1 were ~10.5 GPa and ~14.2 GPa, respectively. This improvement in nanohardness for S1 sample could be the result of combined effects of structural reinforcement and hard ceramic solutes after ion implantation. It is speculated that the presence of a shallow sublayer (~60 nm), composed of amorphous phase and nanocrystalline martensite, favors the improvement of strength and toughness [28,29]. It is noted that the depth to which the hardening phenomenon was observed in S1 (deeper than 120 nm), was more than two times the thickness of the implantation zone (~60 nm, as confirmed in Figure 3). This can be attributed to the long-range effect [13,30].

#### 3.2.2. Tribological Behavior

**Figure 8:**
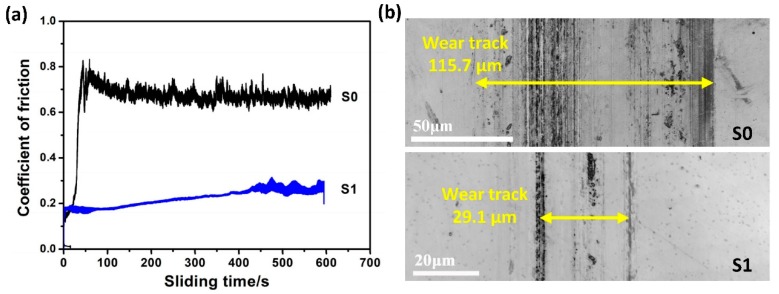
(**a**) Friction coefficients of various samples as a function of time and (**b**) the corresponding morphologies of the wear tracks. The applied load was 2 N.

As shown in Figure 8a, the coefficient of friction (COF) of S0 sample increased rapidly at the initial stage to ~0.8 and then remained constant at about 0.72 for the duration of the test. After the test, severe abrasive wear was found on the contact surface (Figure 8b). However, in the case of S1, the friction process remained at a relatively low level, and the initial COF was 0.18 and it slightly increased to 0.25 until the friction test. The most striking finding is that the wear morphology of S1 surface (Figure 8b) was quite mild and the corresponding wear track width of S1 (29.1 μm) was reduced by about a quarter as compared to that of S0 (115.7 μm). Thus, it can be inferred that ion implantation-induced structural modification and hardening significantly improve the wear resistance of S1.

## 4. Discussion

The above results demonstrate that the ion implantation process effectively improves mechanical and tribological performances of Cronidur30 steel materials, by inducing both unique amorphous/nanocrystalline structures and newly formed ceramic phases. The strengthening mechanism and the relationship between the strengthening effect and mechanical properties are clarified in the discussion below.

### 4.1. Implantation-Induced Structural Evolution

As per the differences in the structures, phases, and components between the non-implanted and implanted samples, it can be concluded that two layers are formed after the implantation process: One is the ion-implanted top layer and the other is the affected layer which originates from long-range effects beneath the ion-implanted layer [13]. According to the EDS-line results in Figure 3, the whole ion-implanted layer is as thick as ~80 nm for S1. This layer can be further divided into two regions. From the TEM results in Figure 2, the top amorphous region of 25 nm is expected to originate from the thermal spike [31] and collision cascade [32] induced by the implantation of high-energy ions into the shallow surface [33]. Beneath this region is an implanted-crystal sublayer, which is formed probably due to the fact that implanted ions scatter and penetrate beyond the amorphous sublayer but do not have enough energies to disorder the original lattice [34,35]. Beneath the ion-implanted layer, the long-range affected layer is formed, in which the implanted ions do not arrive. Even so, the collision energy of these ions leads to refinement. As a result, grain refinement is not only observed in this long-range affected layer, but also in the expanded region next to the implanted-crystal layer. Furthermore, beneath the amorphous layer, the lath martensite grains become finer, undergoing graded refinement in the affected region and forming nanoscale acicular crystals in the implanted-crystal layer (as seen from the TEM results in Figure 2). 

For the constitutions, new ceramic phases are generated in the implanted layer (as seen in XPS results in Figure 4 and Figure 5), which results from precipitation and the formation of substitutional solid solutions for Ti + N atoms. According to the XRD analysis, the ceramic phases including chromium carbides and titanium nitrides are almost amorphous, especially in the case of S1.

### 4.2. Characteristics of Ti + N Co-Implantation

Based on the results presented above, it is reasonable to infer that, to some extent, the length and the constitutions of affected region in dual-elements (Ti + N) implantation differ from that of single-element (Ti) implantation [23]. From the TEM analysis in Figure 2d, the thickness of the amorphous sublayer can be as thick as 25 nm. As compared to the single-element case, the thicker amorphous layer in S1 may be attributed to the combined actions of more intensive cascade bombarding and more foreign atoms incorporated into the bulk, which profoundly disturbs the original lattice. In addition, the total ion-implanted layer in S1 has a thickness of ~80 nm. The implanted-crystal sublayer of S1 is refined and has finer martensite grains, with a greater number of defects such as twins and dislocations being present near the interface (Figure 2c,d). This phenomenon may result from the fact that the higher energy of the dual-elements implantation process triggers more intensive collision cascades and then results in deeper penetration and more defects in the substrate [30,35]. Moreover, as confirmed by the XPS results, the contents of the as-formed ceramic phases in the implanted layer of S1 are much higher than those in S0, even though the categories of chromium carbides and titanium nitrides are the same. One reason may be that additional N ions favor the formation of more titanium nitrides, and the other reason may be that more C atoms (Table 3) defuse to the shallow layer from the bulk, resulting in the formation of more chromium carbides. Moreover, it needs to be highlighted that besides the carbon diffusion from the bulk, ion beam-induced uptake of carbon species and implantation into the sample surface may be also another major source for carbon enrichment in this shallow and amorphous sublayer. As discussed by some researchers [36,37,38,39], traces of gaseous species such as CH_4_, O_2_, CO and hydrocarbons are always present even in high vacuum chamber. During high-energy ion implantation, a significant amount of carbon will be deposited on the material surface or into the near-surface sublayer [38]. The accumulation of carbon and its precipitation form a carbon-rich region with the concentration exceeding the original alloy content, which significantly affects the structural evolution. In general, a thin layer rich in carbon and oxygen is frequently found on the implanted surface [39]. This is also true in the present case that a carbon-rich sublayer is observed in the near-surface region of S1 sample surface with carbon content of 51.13 at.% (Table 2). Based on TEM and XPS results, carbon atoms are expected to diffuse into the amorphous sublayer beneath the sample surface and promote the formation of various carbides. In addition, this carbonaceous sublayer is speculated to be beneficial for enhancing the lubricity of the implanted surface, which is evidenced by the relatively low friction in the initial rubbing stage for the S1 sample (Figure 8a). Therefore, the influence of accidental incorporation of carbon during the ion implantation process should be taken into account during material structure design.

### 4.3. Strengthening Mechanisms

The structural modifications are induced by ion implantation, and meanwhile newly formed ceramic solutes are embedded into the modified structure. It is effective for these two factors to affect the mechanical and tribological properties, which is indicated by the nanoindentation evaluation and friction and wear tests. Furthermore, Ti + N ion implantation is more effective for enhancing the tribomechanical properties as compared to single-element implantation. This phenomenon may be attributed to the structural strengthening and reinforcement of newly formed ceramic solutes. The Ti + N ion implantation process is more energetic, and the ions undergo more intense interactions with the bulk materials. Thus, for the structural modification, Ti + N ion implantation results in deeper amorphous layer, implanted-crystal layer, and long-range affected layer with more crystal defects (dislocations and twins) and gradually-refined lath martensite, which increases the nanohardness and reduces the friction [40,41]. Moreover, the implanted ions can act as sinks to hold back the diffusion of crystal defects and to prevent dislocations and cracks from proliferating. Meanwhile, nanoscale martensite retards the initiation and propagation of cracks [42]. Based on the common theory of wear [16], decreases in the friction coefficient and propagation speed of cracks can effectively improve the wear resistance of the modified liners. In addition, for the reinforcement of ceramic solutes, Ti + N ion implantation also produces more nanocrystalline/amorphous TiN_x_/CrC_x_ phases in the implanted layer. These hardening phases are more beneficial for improving surface strength and lubrication properties [43,44]. Consequently, owing to these two factors, Ti + N dual ion implantation yields much higher nanohardness in the near-surface region, better friction-reducing performance, and wear resistance than nonimplanted sample S0.

## 5. Conclusions

In this study, the effects of Ti + N ion implantation on the nanohardness and tribological properties of Cronidur30 steel were investigated. It was found that a trilamellar structure, composed of an amorphous layer, an implanted-crystal layer and a long-range affected layer, was formed by ion implantation. The Ti + N implantation produced a deep implanted layer with more ceramic phases at the near-surface region. The surface nanohardness of Cronidur30 was seen to be 1.4 times that of pristine surface. The friction level was only one third of the non-implanted sample, and the anti-wear performance was also significantly enhanced. Conclusively, these structural modifications and the in-situ formation of ceramic phases help to improve mechanical and tribological properties.

## Figures and Tables

**Table 1:** Atomic concentrations of S0 at different depths.

at. % Depth/nm	C	N	O	Cr	Fe
0	51.55	1.48	37.40	4.16	5.41
10	4.27	1.46	5.68	15.68	72.90
40	4.01	2.73	5.53	15.75	71.98
60	3.29	1.99	4.85	15.96	73.91

**Table 2:** Atomic concentrations of S1 at different depths.

at.% Depth/nm	C	N	O	Ti	Cr	Fe
0	51.13	11.62	30.81	1.41	0.66	4.37
10	6.50	42.66	8.53	12.71	5.07	24.53
40	5.59	8.69	10.40	9.67	12.07	53.57
60	3.53	6.78	3.92	2.90	13.26	69.62

**Table 3:** The calculation results of grazing incidence X-ray diffraction (GIXRD) spectra.

Samples	Planes	2 Theta/Degree	FWHM/Degree	D/nm
S0	(110)	44.853	1.424	14.5
S1	(110)	44.466	2.686	8.7
